# Predicting the Color Polymorphism of ROY from a Time-Dependent Optimally Tuned Screened Range-Separated Hybrid Functional

**DOI:** 10.1021/acs.jctc.4c00433

**Published:** 2024-06-06

**Authors:** Michal Hartstein, Guy Ohad, Leeor Kronik

**Affiliations:** Department of Molecular Chemistry and Materials Science, Weizmann Institute of Science, Rehovoth 7610001, Israel

## Abstract

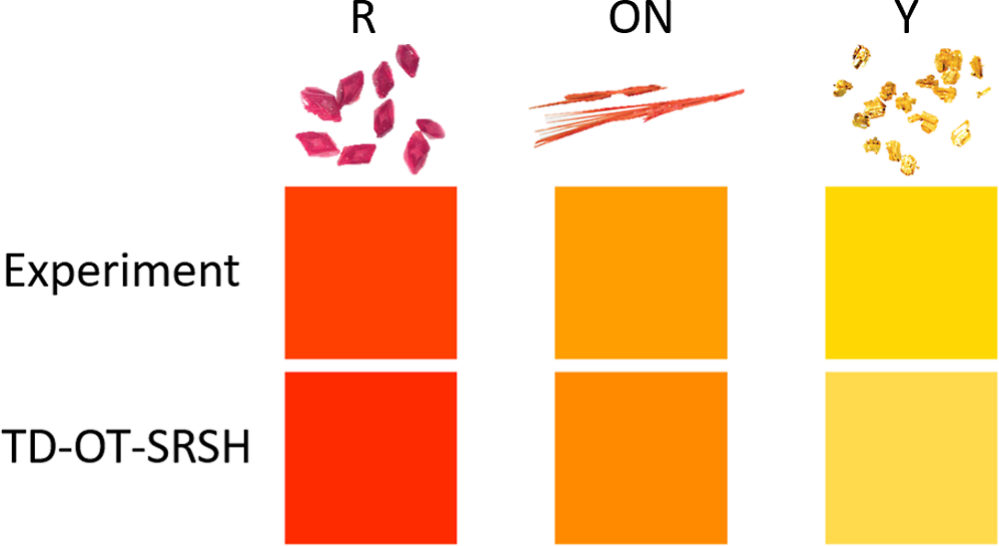

Polymorphism is a well-known property of molecular crystals, which allows the same molecule to form solids with several crystalline structures that can differ significantly in physical properties. Polymorphs that possess different optical absorption properties in the visible range may exhibit different perceived colors, a phenomenon known as color polymorphism. One striking example of color polymorphism is given by 5-methyl-2-[(2-nitrophenyl)amino]-3-thiophenecarbonitrile, known as ROY for its red–orange–yellow colors. First-principles prediction of color polymorphism may help in polymorph assignment and design but has proven to be challenging. Here, we predict the absorption spectra and simulate the colors of 12 ROY polymorphs using the general, nonempirical method of time-dependent (TD) optimally tuned screened range-separated hybrid (OT-SRSH) functional. For 5 ROY polymorphs with known experimental absorption spectra, we show that the TD-OT-SRSH approach predicts absorption spectra in quantitative agreement with experiment. For all polymorphs, we show that an accurate simulation of the colors is obtained, paving the way to a fully predictive, low-cost calculation of color polymorphism.

## 1 Introduction

Molecular crystals are solids comprising a periodic lattice of molecules, bound together by weak interactions, typically van der Waals and/or hydrogen-bond ones.^1^ Such crystals are at the focus of extensive research owing to their importance in various fields, e.g., pharmaceuticals,^2,3^ energetic materials,^4^ electronic and optoelectronic materials,^5,6^ optically functional biogenic materials,^7^ and more.

**Figure 1:**
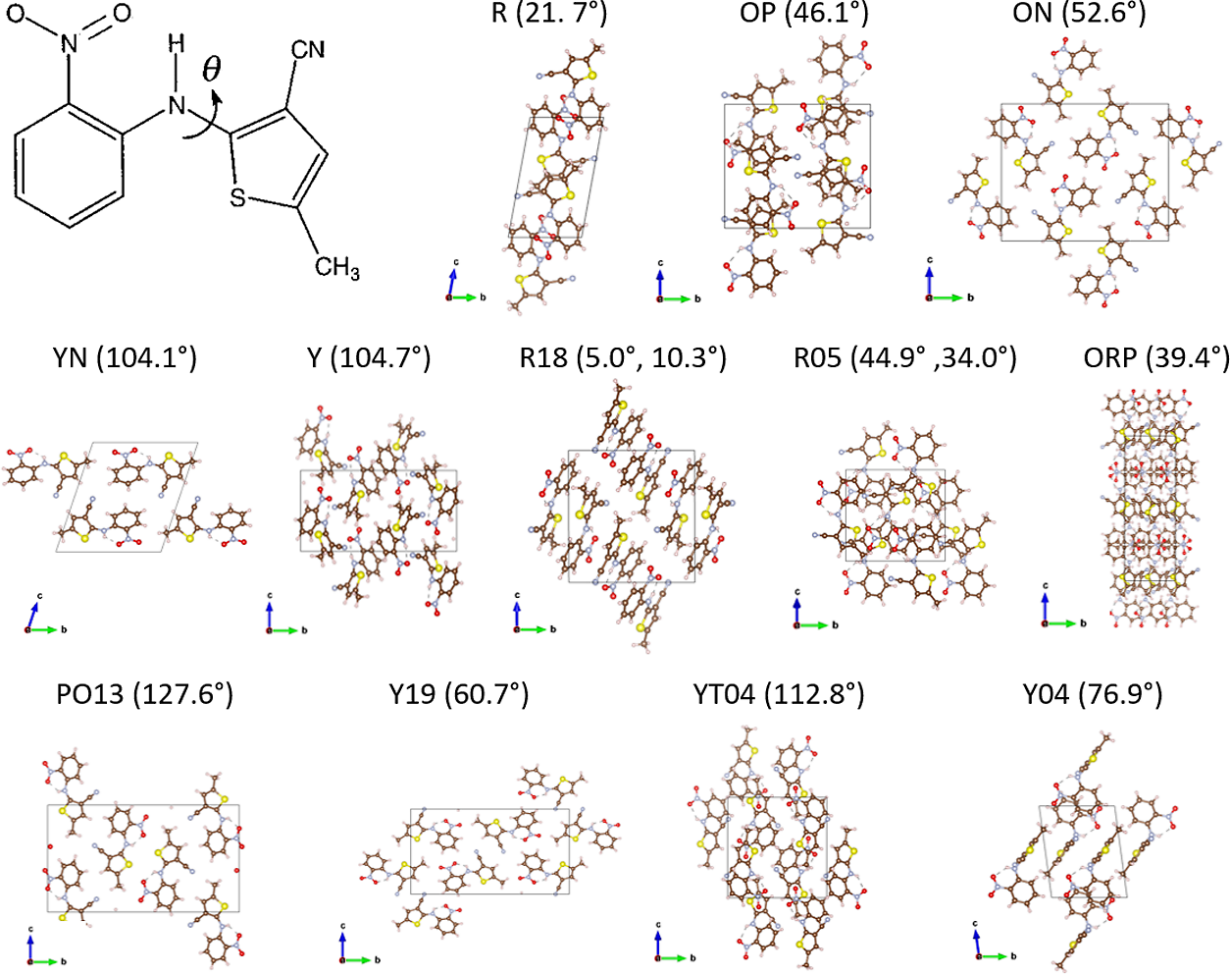
Structures of 12 polymorphs of ROY: R, OP, ON, YN, Y, R18, R05, ORP, PO13, Y19, YT04, and Y04 (see text for details), along with a chemical sketch of the ROY molecule that shows the torsional angle, θ, and its value for each polymorph.

A significant and well-known property of molecular crystals is polymorphism, which allows the same molecule to form solids with many different crystalline structures that may differ significantly in their physical properties.^8^ One important property that in general may vary between different polymorphs is their optical absorption spectrum.^9−11^ When polymorphs exhibit different optical absorption in the visible range, this translates into different perceived colors and is therefore known as color polymorphism,^12^ with significant consequences in polymorph identification and in applications ranging from pigments to sensors.^13,14^ One particularly striking example of color polymorphism is afforded by the organic compound 5-methyl-2-[(2-nitrophenyl)amino]-3-thiophenecarbonitrile, as shown in Figure 1. It is often called ROY because it crystallizes in 13 known crystalline structures^15^ that exhibit a red, orange, or yellow color, where the color variation is correlated with the torsional angle, θ, between the amine and thiophene moieties (see Figure 1).^16^ Polymorphs with more planar ROY molecules (θ values closer to 0 or 180°) tend to be more red, while polymorphs with more twisted ROY molecules (θ values closer to 90°) tend to be more yellow.

Clearly, theoretical prediction of color polymorphism from first-principles—in the sense of computing a perceived color from a given structure without any additional experimental input—can help in polymorph assignment and ultimately in polymorph design, but it is highly challenging.^17^ Time-dependent density functional theory (TDDFT) is a well-established method for prediction of optical absorption in molecular systems.^18^ However, TDDFT using conventional approximate functionals is usually not accurate enough for a quantitative prediction of absorption in solids.^19^ Specifically for ROY, Prentice and Mostofi have shown that TDDFT based on the well-known generalized-gradient approximation of Perdew, Burke, and Ernzerhof (PBE)^20^ yields a qualitatively incorrect color prediction. Instead, they have suggested a correction scheme based on spectral warping according to a comparison of PBE and hybrid functional calculations in ROY dimers.^21^

An alternative, general approach can be found in TDDFT based on an optimally tuned screened range-separated hybrid (OT-SRSH) functional. This method has been suggested as a systematic means of predicting optical absorption in molecular crystals^22^ and has been applied successfully to a variety of molecular solids.^23−27^ However, the correct prediction of the colors of ROY requires an unusually stringent accuracy because a blue shift or red shift of only 0.1 eV may change the perceived color from orange to yellow or red, respectively. It therefore remains to be seen whether the OT-SRSH approach is sufficiently accurate for color prediction of ROY in particular and of molecular crystals in general.

In this article, we address this question by using time-dependent (TD) OT-SRSH to predict the optical absorption spectrum and color of five ROY polymorphs for which the spectrum is known experimentally, as well as the color of seven more polymorphs which have been characterized structurally and exhibit a known color. We find that the TD-OT-SRSH calculations predict the absorption spectra quantitatively and accurately portray the actual appearance of the crystals, thereby potentially opening the door to general-purpose prediction of color polymorphism.

## 2 Methods

### 2.1 ROY Polymorphs

Below we present a detailed comparison between theory and experiment for the absorption spectra and simulated colors of five polymorphs of ROY: R (red),^28,29^ OP (orange plate),^29^ ON (orange needle),^28,29^ YN (yellow needle),^29^ and Y (yellow).^28,29^ Predicted absorption spectra and simulated colors are also given and compared to the experimental colors for the following seven additional polymorphs: R18 (red 2018),^30^ R05 (red 2005),^31,32^ ORP (orange–red plate),^29^ PO13 (pumpkin–orange 2013),^33,34^ Y19 (yellow 2019),^34^ YT04 (Y04 transformed),^35^ and Y04 (yellow 2004).^35,36^ An additional polymorph, RPL (red plate),^37^ was not considered in this work owing to the very large unit cell of its proposed structure.^38^ For each of the polymorphs, we use the experimental structure without further geometry optimization in order to maintain the torsional angle that controls the color and to assess the accuracy of the density functional without possible compounding errors from the structural prediction [generally speaking, optimally tuned range-separated hybrid (RSH) functionals typically result in geometries similar to those obtained from global hybrid functionals, both with^39,40^ and without^41,42^ dispersive corrections].

### 2.2 OT-SRSH Functional

In general, RSH functionals partition the Coulomb repulsion into long-range (LR) and short-range terms, with different approximations for each component.^43,44^ In particular, the SRSH functional used in this work is based on the identity^45^
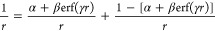
1where *r* is the interelectron coordinate, γ is a range-separation parameter, and α, β are adjustable parameters. The exchange energy owing to the two terms in eq 1 is approximated differently, with the first term treated via exact (Fock) exchange (xx) and the second one via semilocal exchange (SLx). The exchange energy is then expressed as
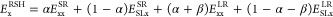
2α and β control the limiting fraction of exact exchange used, which tends to α for *r* → 0 and to α + β for *r* → ∞. γ controls the range at which each of the two terms dominates.

In this study, we use the range-separated version^46,47^ of the PBE exchange functional^20^ to treat the SLx components, along with full PBE semilocal correlation. We use α = 0.2 throughout, which provides an appropriate balance between exchange and correlation, already shown to be useful for small organic molecules.^25,48−51^ The value of β is then determined by demanding the correct asymptotic behavior of the exchange–correlation potential.^23,48,52,53^ For molecules, the ∼1/r asymptotic behavior^54,55^ is enforced by setting α + β = 1.^49,56^ For solids, we account for dielectric screening that changes the asymptotic potential to ∼1/(εr), where ε is the scalar dielectric constant, by setting α + β = 1/ε. The latter case is known as the screened RSH (SRSH) approach.^48^

In an optimally tuned (OT) RSH functional, the range separation parameter, γ, is determined nonempirically, per system, by obeying the ionization potential (IP) theorem.^52,57^ The IP theorem^54,55,58^ states that for the exact functional, the IP computed from total energy differences, *E*(*N* – 1) – *E*(*N*), between the (*N* – 1)-electron and *N*-electron systems, is equal and opposite to the highest occupied molecular orbital (HOMO) eigenvalue of the *N*-electron system, ε_HOMO(*N*)_. We therefore seek an optimal γ for the SRSH functional such that
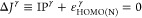
3

In this study, the tuned γ was obtained for the isolated molecule, taken directly from the pertinent crystal structure without optimization. This was performed using NWchem v.6.8.1^59^ with the cc-pVQZ basis set.^60,61^ The dielectric constant of the crystal was calculated using the PBE functional, with β in the SRSH functional adjusted accordingly. We verified for selected systems that there is no meaningful change in the results if ε is recomputed based on the final SRSH functional.

TDDFT calculations were used with the nonempirically determined SRSH parameters^22,25,62^ to calculate optical absorption spectra. For each polymorph, we extracted the calculated absorbance in a specific direction as reported for the measured spectra: (111)R, (1̅01)OP, (011)ON, (010)Y, and the average spectra from all directions for YN (which is polycrystalline) and for all other polymorphs where only the color is known. All (TD)DFT calculations for the molecular solids were performed using a modified version of the Vienna ab initio simulation package 6.3.1 code,^63^ using a plane-wave cutoff of 1000 eV, with *k*-point grids reported in Section I of the Supporting Information. To obtain realistic, smoothed spectra, we applied a Gaussian broadening of 0.25 eV to the raw TDDFT excitation energies, weighted by the relevant oscillator strength. All spectra were normalized such that absorbance values ranged from 0 to 1.

### 2.3 Color Simulation from Absorbance Spectra

Color simulations can be performed in a variety of ways, each with the aim of mimicking how the human eye subjectively perceives color based on an objective light spectrum. We emphasize that color perception is a complicated question in itself and depends on many factors, including the level of illumination. Here, we use the CIE 1931 color matching functions to convert the spectra to the red–green–blue format.^64,65^ The purpose of this particular choice is to provide an estimate of how well the calculations predict color relative to experiment. To ascertain that it is appropriate, we verified that applying this conversion scheme to the experimentally measured absorbance spectra yields colors close to those observed by the unaided eye. Further details are given in Section II of the Supporting Information.

## 3 Results and Discussion

As explained above, we first tuned the range-separation parameter, γ, for the molecular geometry obtained from the five polymorphs of R, OP, ON, Y, and YN. Demanding a precision of Δ*J* < 0.04 eV in eq 3, we found that all tuned values were almost the same, with minor differences of less than 0.02 Å^–1^. In other words, the range-separation parameter, γ, is hardly sensitive to the molecular torsional angle, θ. We therefore selected a value of γ = 0.3 Å^–1^ as a common one for all polymorphs. An example of the deleterious effect of using a nonoptimal γ with large Δ*J* values on the predicted color of the R polymorph is given in Section III of the Supporting Information, thereby establishing the importance of the optimal tuning procedure. Likewise, we found that the scalar dielectric constant, ε, was very similar for all five polymorphs, with differences below 0.3, which are known to have a very minor effect on the optical absorption spectrum.^25^ We therefore set the dielectric constant value to 3.3 for all polymorphs.

**Figure 2:**
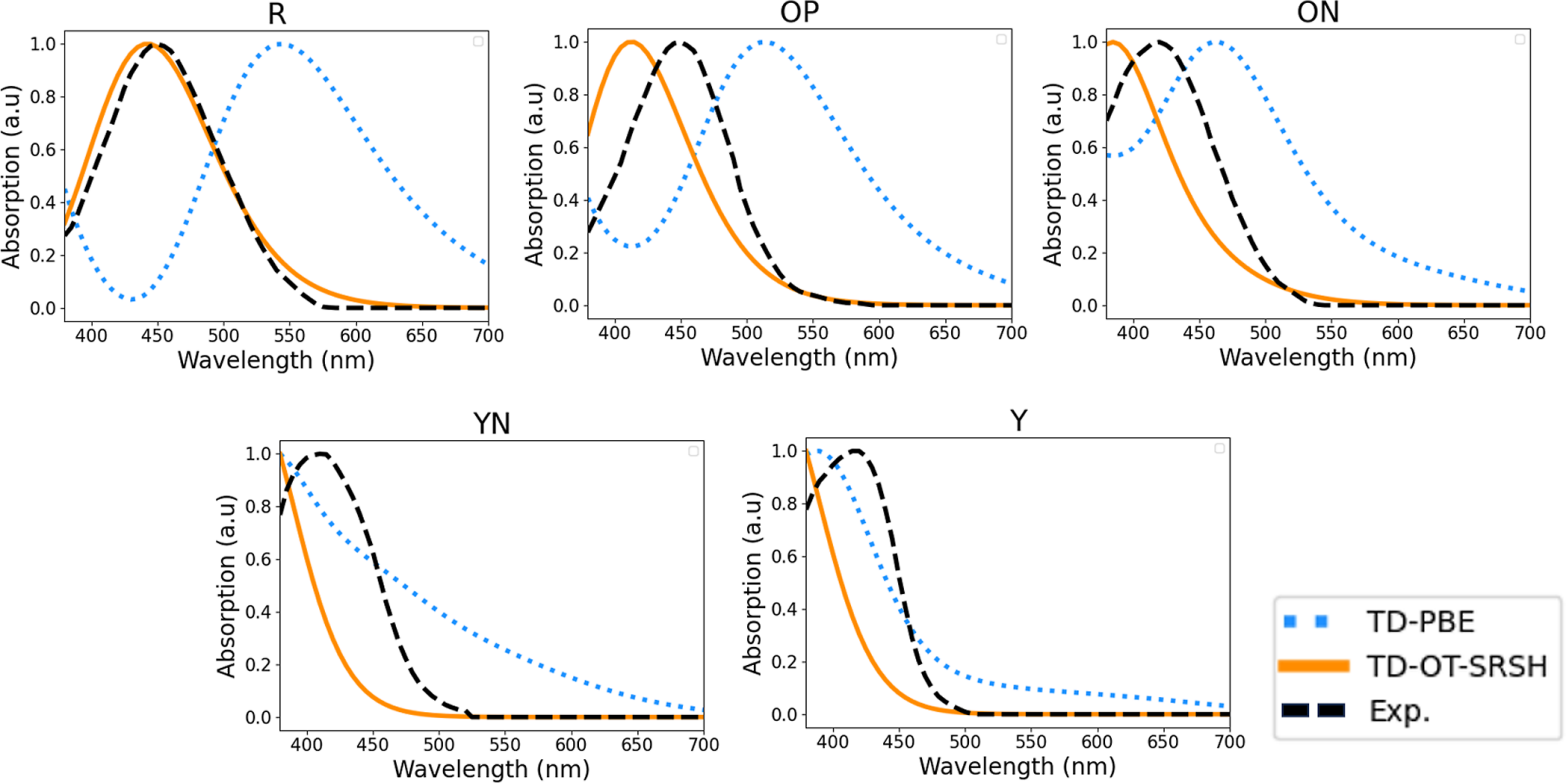
Experimentally measured absorption spectra (dashed black line), digitized from the work of Yu^10^ and used with permission, compared to calculated absorption spectra (dotted blue line: TD-PBE; orange solid line: TD-OT-SRSH) of the R, OP, ON, YN, and Y polymorphs of ROY.

Figure 2 provides a detailed comparison of the absorption spectra obtained using TD-PBE and TD-OT-SRSH, compared to the experimental spectra measured by Yu,^10^ for the R, OP, ON, YN, and Y polymorphs. The comparison focuses on the visible spectral range, from 380 to 700 nm, which is obviously the one pertinent to color perception. For all polymorphs, the TD-OT-SRSH results match the experimental data well, showing very close excitation peaks, differing by less than 30 nm (or less than 0.2 eV) from experiment and exhibiting overall similar spectral line shapes. In contrast, the TD-PBE results show clear differences from the experimental data, with significant variations in peak excitation energies and/or spectral line shape for all polymorphs except Y, where agreement is likely coincidental.

**Figure 3:**
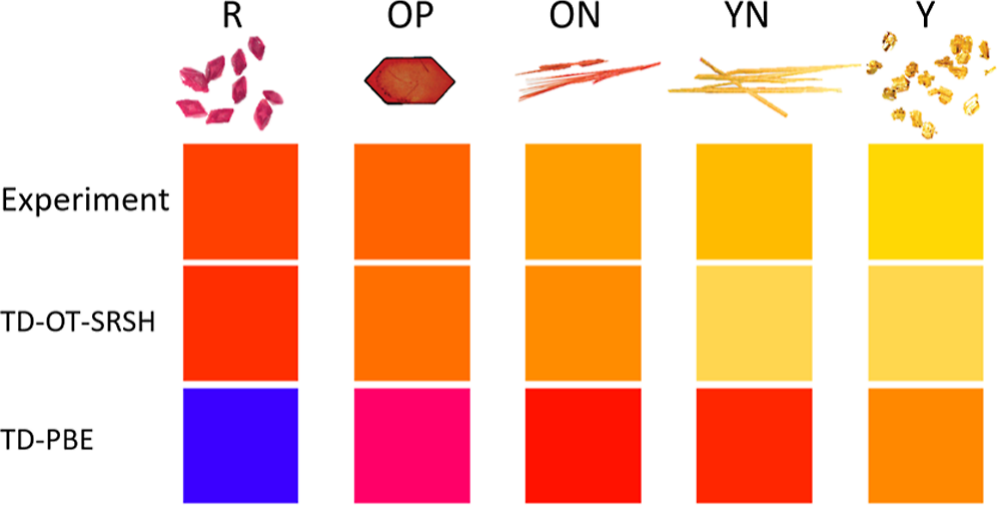
Top: images of the five polymorphs studied, adapted from the work of Yu^16^ and used with permission. Bottom: colors simulated based on the experimentally measured and theoretically calculated absorption spectra shown in Figure 2.

The colors predicted from the absorption spectra given in Figure 2 are presented in Figure 3, along with images of the crystallized polymorphs adapted from the work of Yu. The agreement between the experimental results and the TD-OT-SRSH predictions is very good, highlighting the precision of TD-OT-SRSH in capturing the nuanced color tones of the ROY polymorphs. This accuracy stands out especially when compared to the TD-PBE predictions, which significantly misrepresent the colors of the polymorphs. Specifically, for the R polymorph, both experiment and TD-OT-SRSH predict very similar red tones, while TD-PBE predicts blue. For OP, experiment and TD-OT-SRSH predict similar orange tones, whereas TD-PBE predicts a pink-violet color. For ON, experiment and TD-OT-SRSH predict similar tones of orange, whereas TD-PBE predicts red. For YN, the experiment predicts an orange-yellow tone, TD-OT-SRSH predicts a yellow color, and TD-PBE predicts red. Finally, for Y, experiment and TD-OT-SRSH predict similar yellow tones, whereas TD-PBE predicts orange.

**Figure 4:**
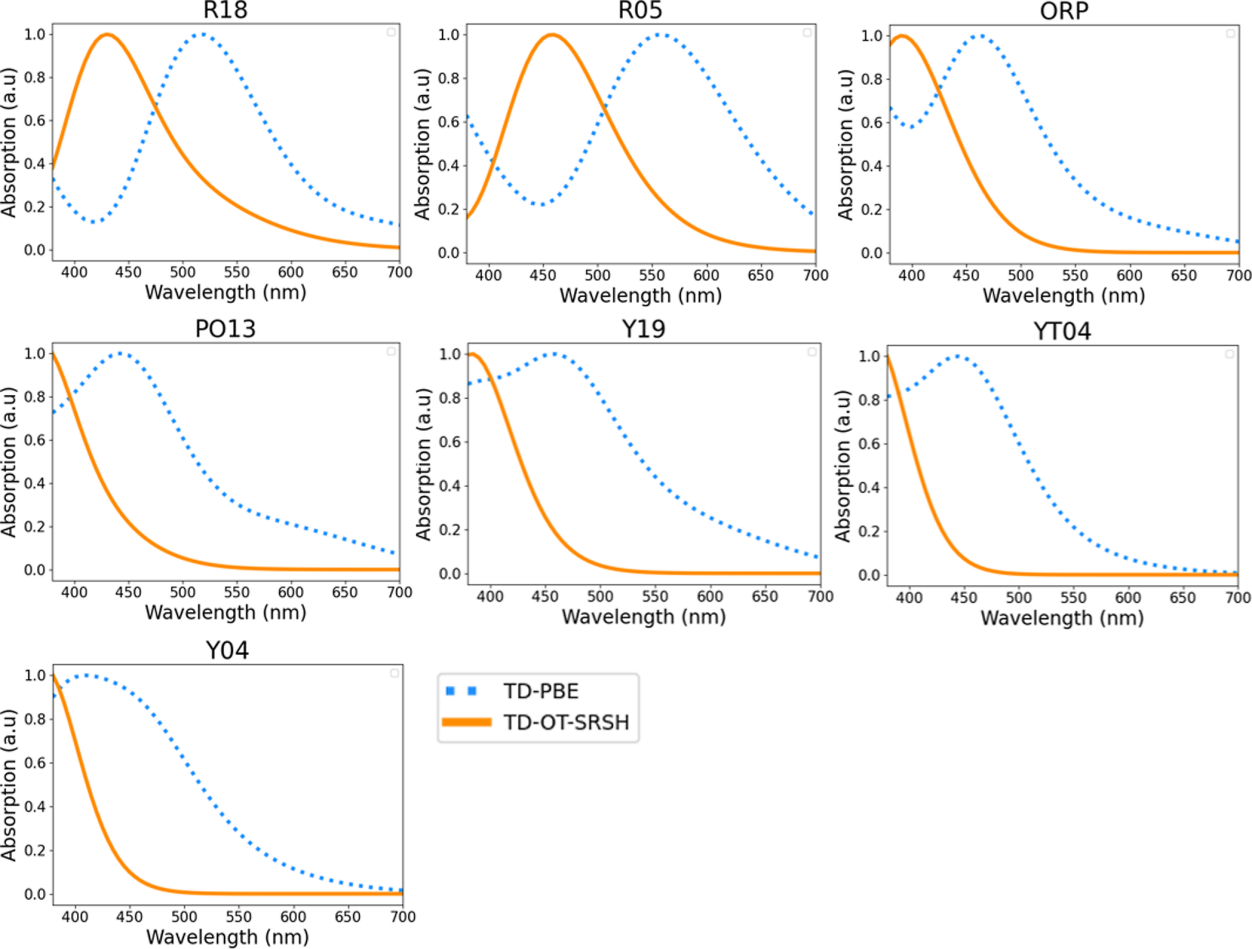
Calculated absorption spectra (dotted blue line: TD-PBE; orange solid line: TD-OT-SRSH) of the R18, R05, ORP, PO13, Y19, YT04, and Y04 polymorphs of ROY.

**Figure 5:**
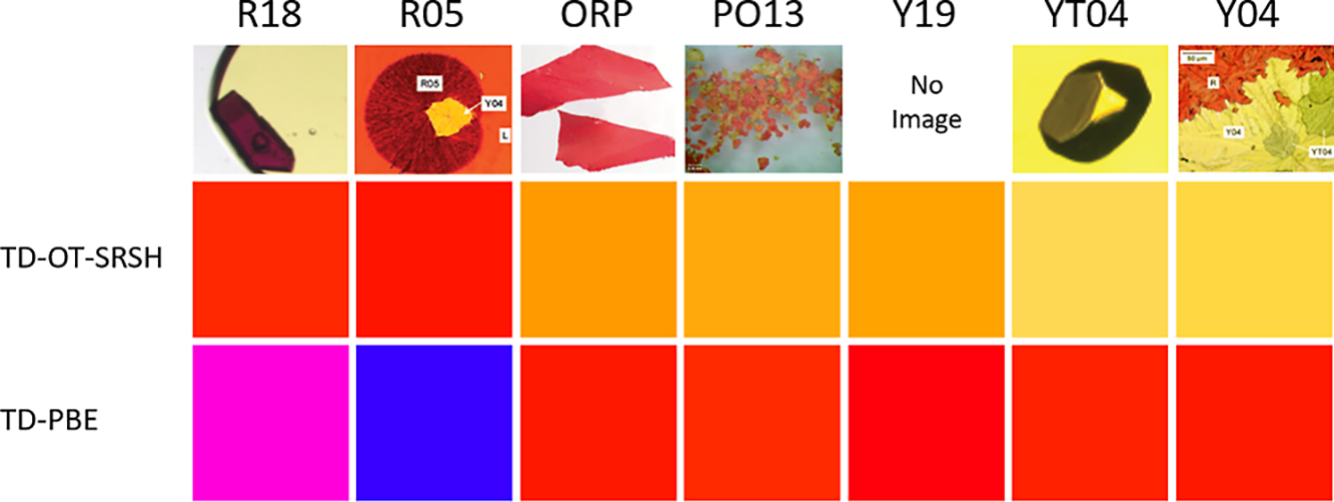
Top: images of six ROY polymorphs: R18 is adapted from ref (30), R05, ORP, YT04, and Y04 from ref (16), and PO13 from ref (33), all used with permission. Bottom: colors simulated based on the computed absorption spectra shown in Figure 4.

Encouraged by the above success, we also predict the absorption spectra and simulated colors, as shown in Figures 4 and 5, respectively, of 7 additional ROY polymorphs. The structures and colors are also well-established for these polymorphs, but to the best of our knowledge, experimental absorption spectra are not available for them. For the R18 polymorph, TD-OT-SRSH predicts red, while TD-PBE predicts a pink-violet color. For R05, TD-OT-SRSH predicts red, while TD-PBE predicts blue. For ORP, TD-OT-SRSH predicts orange, and TD-PBE predicts red. For PO13, TD-OT-SRSH and TD-PBE predict different orange tones. For Y19, TD-OT-SRSH predicts orange, and TD-PBE predicts red. For Y04 and YT04, TD-OT-SRSH predicts yellow, while TD-PBE predicts red. Thus, we find that TD-OT-SRSH also provides good predictions for the tones of these additional polymorphs.

## 4 Conclusions

In conclusion, we applied the OT-SRSH approach to the nonempirical calculation of optical spectra and prediction of color polymorphism of ROY structures. The method starts with an accurate and predictive calculation of the gas-phase electronic structure, through optimal tuning of a range-separation parameter, and proceeds with the application of LR dielectric screening. For ROY, both the range-separation and the asymptotic screening were found to be nearly the same for the R, OP, ON, YN, and Y polymorphs, indicating that the chemical nature of the system is captured by a uniform set of parameters. The obtained results have been compared with experimental absorption spectra, and agreement has been found to be very good to excellent throughout. Moreover, we have demonstrated that accurate colors can be obtained with the same parameters for all polymorphs studied, paving the way to a fully predictive, low-cost calculation of color polymorphism.

## Supporting Information Available

*K*-point grids used for all calculations of the ROY polymorphs; further details on the color simulations from absorbance spectra used in this study; and SRSH color predictions for the R polymorph with different γ and Δ*J* values (PDF)
