# Supplementary material for: Predicting the Color Polymorphism of ROY from a Time-Dependent Optimally Tuned Screened Range-Separated Hybrid Functional
Source: J Chem Theory Comput. 2024 Jun 6;20(13):5510–6. doi: 10.1021/acs.jctc.4c00433 (PMC11238539; doi:10.1021/acs.jctc.4c00433)
Supplement: Supplementary file 1 — ct4c00433_si_001.pdf [file ct4c00433_si_001.pdf]

Supporting Information: Predicting the color polymorphism of ROY from a time-dependent optimally-tuned Screened range-separated hybrid functional

Michal Hartstein, Guy Ohad, and Leeor Kronik*

Department of Molecular Chemistry and Materials Science, Weizmann Institute of Science, Rehovoth 7610001, Israel

E-mail: leeor.kronik@weizmann.ac.il

I. K-point grids

Table S1: K-point grids used for all calculations of the ROY polymorphs.

Polymorph	K-point grid
R	3 3 2
OP	3 2 2
ON	4 1 1
YN	3 2 2
Y	2 1 2
R18	4 3 3
RO5	2 2 2
ORP	1 2 1
PO13	5 1 2
Y19	6 1 2
YTO4	3 2 2
YO4	3 3 3

II. Color simulations from absorbance spectra

To simulate the color of the polymorphs from their absorbance spectra $A(\lambda)$, given in the normalized spectra of Figure 2 in the main text and Figure S1 in the SI, we follow the method of tristimulus colorimetry theory.[1–3] We first calculate the transmitted light intensity $I(\lambda)$ using

$$I(\lambda) = I_0(\lambda) \cdot \exp(-A(\lambda) \cdot t) \tag{S1}$$

where $I_0(\lambda)$ is the illuminant, using the CIE standard D65 illuminant (a common choice to approximate daylight[4]), and t is the normalized thickness of the sample. To ensure consistency, we employ a value of $t = 10$ for both experimental and calculated spectra. This choice provides realistic color rendering and is helpful for comparison between the simulated colors.

To mimic the response of the human cone cells to the transmitted light, we then use the tristimulus color-matching functions $\bar{x}(\lambda)$, $\bar{y}(\lambda)$, and $\bar{z}(\lambda)$, as defined by the CIE 1931 standard, to obtain the color indices X, Y, and Z:

$$\begin{pmatrix} X \\ Y \\ Z \end{pmatrix} = \int I(\lambda) \begin{pmatrix} \bar{x}(\lambda) \\ \bar{y}(\lambda) \\ \bar{z}(\lambda) \end{pmatrix} d\lambda. \tag{S2}$$

The integrals are computed over the visible range (380-700 nm). Then, X, Y, and Z are normalized by multiplying each with $\frac{1}{X+Y+Z}$, in order to obtain the chormaticity and discarding the information about the brightness of the light. Finally, the normalized X, Y, and Z are converted to RGB values using a transformation matrix appropriate for the color system sRGB.[1,5]

III. SRSH color predictions for the R polymorph with different γ and ΔJ values

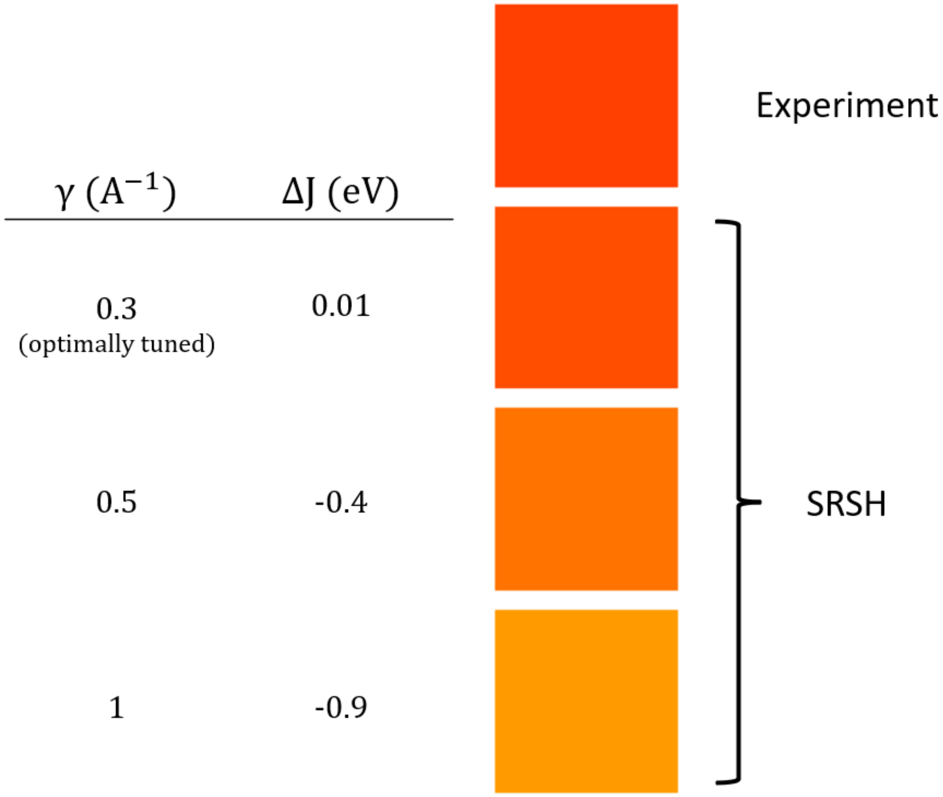

Figure S1: Color simulations for the R polymorph, created from absorption spectra calculated using the SRSH functionals, all with α=0.2, ϵ=3.3, and different γ values, resulting in different ΔJ values. Color simulation was obtained using the same method in the main text with a Gaussian broadening of 0.2 eV. As ΔJ increases in absolute value, the predicted color becomes less similar to the color predicted for experiment.

References

(1) Schanda, J. *Colorimetry: understanding the CIE system*; John Wiley & Sons, 2007.

(2) Zuehlsdorff, T.; Haynes, P.; Payne, M.; Hine, N. Predicting solvatochromic shifts and colours of a solvated organic dye: The example of nile red. *J. Chem. Phys.* **2017**, *146*.

(3) Ge, X.; Timrov, I.; Binnie, S.; Biancardi, A.; Calzolari, A.; Baroni, S. Accurate and inexpensive prediction of the color optical properties of anthocyanins in solution. *J. Phys. Chem. A* **2015**, *119*, 3816–3822.

(4) CIE - International Commission on Illumination, see also www.cie.co.at.

(5) Anderson, M.; Motta, R.; Chandrasekar, S.; Stokes, M. Proposal for a standard default color space for the internet—srgb. Color and imaging conference. 1996; pp 238–245.
